# A Novel Floating *In Situ* Chewable Gel System for Curcumin Delivery with Potential Application in Obesity Management

**DOI:** 10.3390/gels12040286

**Published:** 2026-03-29

**Authors:** Saravoot Pumjan, Rachanida Praparatana, Ousanee Issarachot, Kantiya Fungfoung, Ruedeekorn Wiwattanapatapee

**Affiliations:** 1 Department of Pharmaceutical Technology, Faculty of Pharmaceutical Sciences, Prince of Songkla University, Hatyai 90112, Songkhla, Thailand; saravoot.p@psu.ac.th (S.P.); kantiya.1537@gmail.com (K.F.); 2 Faculty of Medical Technology, Prince of Songkla University, Hatyai 90112, Songkhla, Thailand; rachanida.p@psu.ac.th; 3 Department of Pharmacy Technician, Faculty of Public Health and Allied Health Sciences, Sirindhorn College of Public Health Trang, Kantang 92110, Trang, Thailand; ousaneemu@gmail.com; 4 Phytomedicine and Pharmaceutical Biotechnology Excellence Center, Faculty of Pharmaceutical Sciences, Prince of Songkla University, Hatyai 90112, Songkhla, Thailand

**Keywords:** curcumin, solid dispersion, chewable gel, gastroretention, obesity, anti-inflammatory

## Abstract

Curcumin exhibits potent anti-obesity and anti-inflammatory activities; however, its therapeutic application is limited by poor aqueous solubility and low oral bioavailability. A curcumin-loaded chewable gel was developed to transform into an in situ gastric gel upon contact with gastric fluid after mastication. Curcumin solid dispersions (CUR-SDs) were prepared with Eudragit^®^ EPO (1:1–1:7, *w*/*w*) using the solvent evaporation method. The optimized formulation (1:3) markedly enhanced solubility and dissolution in acidic medium (0.1 N HCl, pH 1.2) compared with crystalline curcumin and physical mixtures. The optimized CUR-SD was subsequently incorporated into chewable gels composed of sodium alginate and κ-carrageenan, with calcium carbonate as a gas-forming agent. The formulations formed buoyant matrices under acidic conditions, exhibiting floating lag times of 21–215 s and sustaining drug release for up to 8 h. Increasing polymer content improved mechanical strength and modulated release kinetics. Among the tested formulations, F7 achieved the optimal balance between texture properties, floating behavior, and controlled-release performance. In LPS-stimulated RAW264.7 macrophages, curcumin, CUR-SD, and F7 showed comparable and potent anti-inflammatory activity (IC_50_ = 4.12–4.84 µg/mL), outperforming indomethacin. In 3T3-L1 adipocytes, F7 significantly reduced lipid accumulation (~47%) in a concentration-dependent manner. These findings demonstrate that this transformable chewable in situ gelling platform is a promising gastroretentive strategy for improving the oral therapeutic efficacy of poorly soluble bioactive compounds for anti-obesity applications.

## 1. Introduction

Obesity is a complex chronic disease and a major risk factor for metabolic complications such as impaired insulin sensitivity, type 2 diabetes mellitus, lipid abnormalities, and metabolic dysfunction-associated fatty liver disease. Current pharmacological therapies, including appetite suppressants and lipolysis modulators, can produce meaningful weight loss but are limited by side effects that reduce long-term adherence [1,2]. These limitations have fueled interest in natural alternatives. Functional foods, herbal medicines, and plant-derived bioactive compounds are widely regarded as safer and more tolerable options [3]. Numerous phytochemicals have shown potential anti-obesity effects through mechanisms such as inhibition of adipogenesis, modulation of lipid metabolism, enhancement of energy expenditure, and appetite regulation. Notable examples include quercetin [4], epigallocatechin gallate from green tea [5], garcinia extract [6], proanthocyanidin from grape seed extract [7], capsaicin from chili [8], glucomannan [9], resveratrol [10], anthocyanin from roselle [11], and curcumin from turmeric [12].

Curcumin, the predominant bioactive polyphenol isolated from *Curcuma longa*, has been widely investigated for its metabolic regulatory, antioxidant, and anti-inflammatory effects. Accumulating evidence indicates that curcumin attenuates obesity by influencing multiple molecular targets associated with adipogenesis, lipid metabolism, and chronic inflammation [13]. It enhances energy expenditure primarily through AMPK activation, modulates adipogenic regulators such as PPARγ and C/EBPs, and promotes fatty acid oxidation while limiting lipid accumulation [14]. In addition, curcumin suppresses inflammatory signaling pathways, including NF-κB, leading to reduced expression of cytokines such as TNF-α, MCP-1, and PAI-1. Experimental models demonstrate decreased body weight gain, improved insulin responsiveness, and reduced hepatic lipogenesis, whereas clinical studies report modest but significant improvements in BMI, body weight, and waist circumference in overweight and obese subjects [14,15]. Nevertheless, the therapeutic translation of curcumin remains challenging due to its poor oral bioavailability, which stems from limited aqueous solubility, rapid biotransformation, and suboptimal gastrointestinal absorption. To address these limitations, diverse formulation approaches such as lipid-based carriers, cyclodextrin inclusion complexes, polymeric micelles, nanocrystals, and solid dispersions have been explored to enhance its solubility and systemic availability [16].

Amorphous solid dispersions (ASDs) represent an effective strategy to enhance the solubility in aqueous media and dissolution profile of poorly soluble drug candidates. Their superiority arises from improved wettability, reduced effective particle size, increased surface area through porous structures, and suppression of drug crystallinity [17,18]. Eudragit^®^ EPO is a cationic copolymer composed of methyl methacrylate, 2-(dimethylamino)ethyl methacrylate, and n-butyl methacrylate in a 1:2:1 ratio. Owing to the presence of ionizable tertiary amine groups, it readily dissolves under acidic conditions (pH < 5). Its pH-responsive solubility, gastric compatibility, and taste-masking capability make it a widely used carrier for ASD formulations intended for stomach-targeted drug delivery [19].

Oral in situ gelling systems are gastroretentive platforms that undergo sol–gel transition in the gastric environment, forming a floating matrix that prolongs residence time and enables sustained drug release. These systems typically employ ion-responsive polymers (e.g., alginate, pectin, or gellan gum) in combination with gas-generating and calcium-releasing agents to induce gelation and buoyancy [20,21]. A recent study demonstrated a liquid in situ gel containing hydroxycitric acid from *Garcinia atroviridis* with anti-lipogenic activity [6]. However, liquid formulations often require substantial amounts of alkaline excipients, which may compromise the stability of alkali-sensitive drugs and promote degradation. Converting such systems into solid dosage forms may therefore enhance stability and handling. Chewable gels represent a molded, hydrocolloid-based solid dosage form that combines structural integrity with improved patient acceptability and convenience compared to bulky liquid systems. Furthermore, formulations based on κ-carrageenan in combination with sodium alginate are considered promising for obesity management, as their gelation properties may promote satiety. Produced without compression, these gel-based tablets simplify manufacturing while providing a versatile platform for pharmaceutical and nutraceutical applications [22].

The objective of this study was to develop and characterize a novel chewable gel formulation incorporating a curcumin-based amorphous solid dispersion, designed to generate a floating in situ gel for obesity management. The biological performance of the formulation was evaluated through in vitro anti-inflammatory activity in RAW264.7 macrophages and anti-obesity efficacy by assessing lipid accumulation inhibition in 3T3-L1 adipocytes.

## 2. Results and Discussion

### 2.1. Physicochemical Characterization of Curcumin Solid Dispersions

#### 2.1.1. Solubility of Curcumin Solid Dispersions (CUR-SDs)

**Figure 1:**
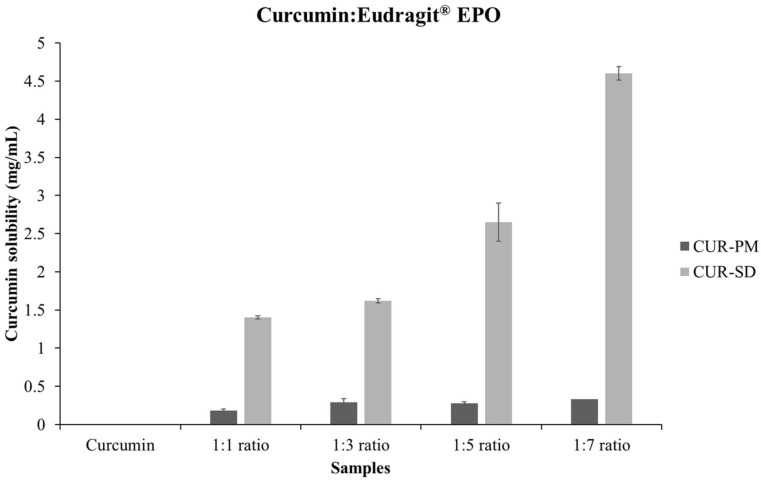
A comparison of curcumin solubility between solid dispersions and physical mixtures.

Curcumin exhibits poor aqueous solubility, which limits its oral bioavailability. The solubility of free curcumin in 0.1 N HCl (pH 1.2) was approximately 0.5 µg/mL, indicating the need for an effective solubility-enhancing system. Physical mixtures (CUR-PMs) of curcumin and Eudragit^®^ EPO at weight ratios of 1:1, 1:3, 1:5, and 1:7 showed solubility values below 0.3 mg/mL (Figure 1), suggesting that simple blending was insufficient to significantly improve curcumin solubility. In contrast, CUR-SDs markedly enhanced solubility. All CUR-SD formulations exhibited approximately a 1500-fold increase in solubility compared to free curcumin, reaching about 0.92 mg/mL.

This substantial improvement is likely due to intermolecular interactions between curcumin and Eudragit^®^ EPO. Specifically, hydrogen-bonding interactions involving the phenolic hydroxyl moieties of curcumin and the cationic functional groups of the polymer may facilitate the formation of water-soluble complexes [23]. These findings are in agreement with previous studies on curcumin–Eudragit^®^ E100 solid dispersions and related Cur–EPO systems [24,25]. The use of Eudragit^®^ EPO, a hydrophilic and pH-responsive polymer, enables the formation of a soluble curcumin form under acidic conditions, making it a suitable carrier for stomach-targeted drug delivery applications.

#### 2.1.2. Dissolution Profiles of CUR-SDs

**Figure 2:**
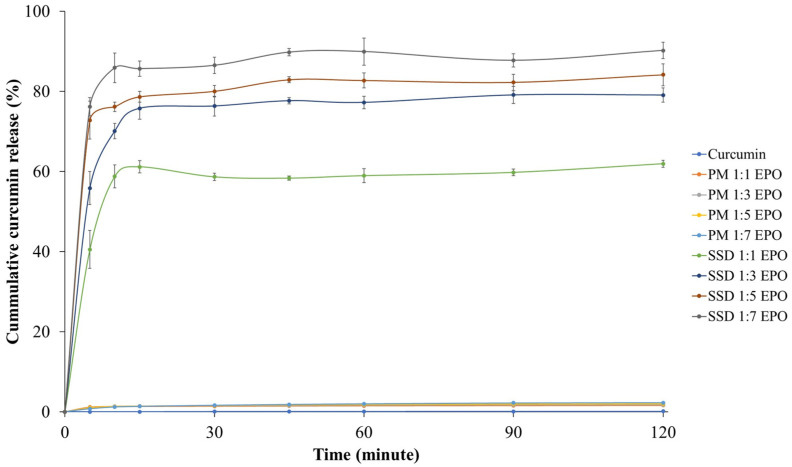
Dissolution release profile of free curcumin, CUR-PMs, and CUR-SDs.

The dissolution profiles of free curcumin, CUR-PMs and CUR-SDs are shown in Figure 2. The release of free curcumin was less than 0.1% *w*/*v* after 2 h, which can be attributed to its poor aqueous solubility. In comparison, the CUR-PMs showed only a slight increase in dissolution, reaching approximately 1–2% *w*/*v* within the same period.

The solid dispersion technique clearly demonstrated that incorporating Eudragit^®^ EPO at various drug-to-carrier ratios significantly improved the dissolution of curcumin. The CUR-SD formulations achieved cumulative release percentages of 61.95%, 79.06%, 84.14%, and 90.21% at drug-to-carrier ratios of 1:1, 1:3, 1:5, and 1:7, respectively (Figure 2). Among the evaluated formulations, the CUR-SD ratio of 1:3 was selected for further development into an in situ chewable gel due to its favorable solubility and dissolution characteristics.

#### 2.1.3. X-Ray Powder Diffraction (XRPD) Analysis

**Figure 3:**
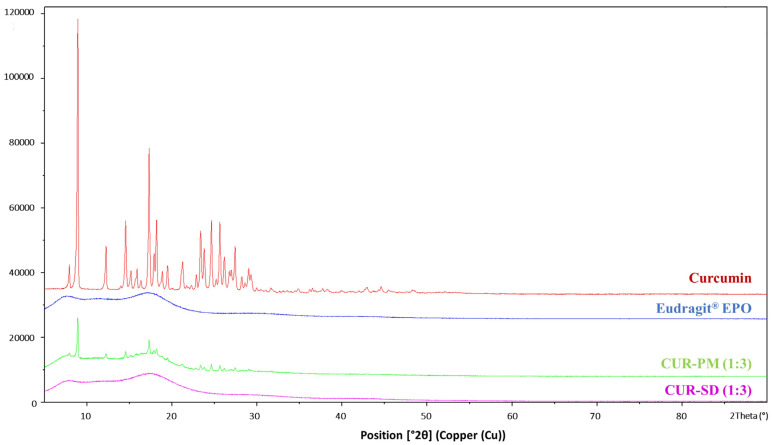
XRPD characteristics of curcumin, Eudragit^®^ EPO, CUR-PM (1:3), and CUR-SD (1:3).

The physical state of curcumin after incorporation into the Eudragit^®^ EPO polymer matrix was evaluated, and its crystallinity was assessed using XRPD, as shown in Figure 3. Dominant diffraction peaks at 2θ values of 8.91°, 12.23°, 14.52°, 17.31°, 18.19°, 23.36°, 24.64°, and 25.63° confirmed the crystalline nature of free curcumin. In contrast, the broad halo pattern observed for Eudragit^®^ EPO suggested its amorphous character.

The CUR-PM exhibited the characteristic crystalline peaks of curcumin, indicating the absence of significant interactions during simple blending. However, the diffractograms of the CUR-SD (1:3) formulation revealed a complete disappearance of curcumin’s crystalline peaks, suggesting its successful conversion into the amorphous state (Figure 3). This transformation is likely attributed to the ability of Eudragit^®^ EPO to inhibit crystallization, possibly by restricting the molecular mobility of curcumin through the formation of hydrogen bonding and entrapment within the polymer matrix [24].

#### 2.1.4. FT-IR Spectral Analysis

**Figure 4:**
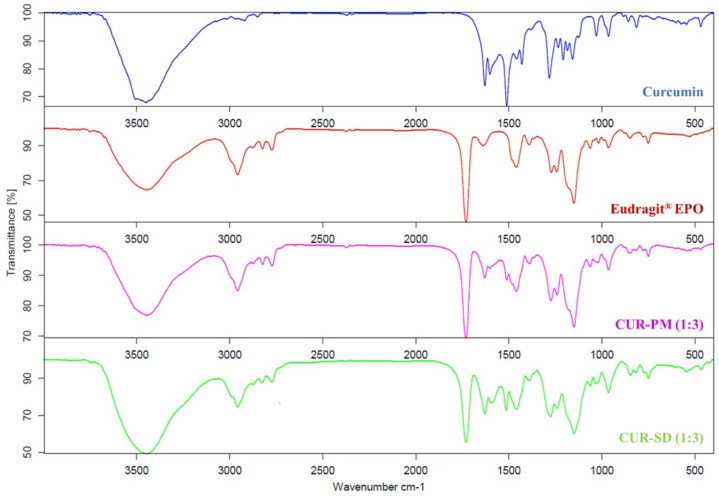
FT-IR spectra of curcumin, Eudragit^®^ EPO, CUR-PM, and CUR-SD.

FT-IR spectroscopy was performed to investigate the molecular compatibility between curcumin and the Eudragit^®^ EPO carrier. The FT-IR spectra of curcumin, Eudragit^®^ EPO, the CUR-PM, and the corresponding CUR-SD are presented in Figure 4.

The FT-IR spectrum of curcumin (Figure 4) exhibited a broad band in the range of 3428–3507 cm^−1^, corresponding to the O–H stretching vibrations of the polyphenolic hydroxyl groups present in its structure. In the case of Eudragit^®^ EPO, characteristic peaks were observed at 1730 cm^−1^ for C=O stretching vibrations (indicative of the carbonyl ester group), a strong absorption band at 1150 cm^−1^ corresponding to C–O stretching, and a medium-intensity broad band at 3447 cm^−1^ attributed to N–H stretching vibrations of amine functionalities [24]. The FT-IR spectra of the CUR-PM (1:3) samples exhibited characteristic peaks of both curcumin and Eudragit^®^ EPO, suggesting a lack of significant intermolecular interactions between the two components. In contrast, the FT-IR spectrum of the CUR-SD (1:3) showed the vanishing of the O–H stretching signal of curcumin at 3448 cm^−1^, the N–H stretching peak at 3448 cm^−1^, and the C=O stretching band of Eudragit^®^ EPO at 1730 cm^−1^, as shown in Figure 4. These spectral changes indicate the formation of strong intermolecular hydrogen bonds between the hydroxyl moieties of curcumin and the positively charged functional groups of polymers. Similar interactions have been previously reported for CUR-SD entrapped in Eudragit^®^ EPO matrix [23,25], and are considered responsible for the transformation of curcumin from its crystalline to amorphous form, leading to enhanced solubility.

### 2.2. Physical Properties of Gummy Jellies

#### 2.2.1. Appearance of the Formulations

**Figure 5:**
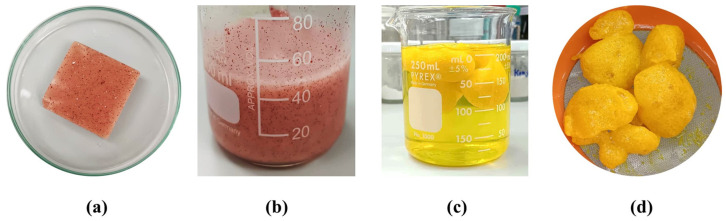
Physical appearances of a chewable gel containing CUR-SD (**a**), reconstituted liquid gel (**b**), floating in situ gels in acidic medium at pH 1.2 (**c**), and yellow-formed gels (**d**).

The CUR-SD-loaded chewable gel was formulated in a square shape using KC and SA as the base formulation. Small pink particles were visibly dispersed throughout the gel (Figure 5a), representing CUR-SD incorporated with Eudragit^®^ EPO, a pH-responsive polymer [19]. Eudragit^®^ EPO effectively masks the undesirable aftertaste of curcumin [23,26]. It mimics the action of chewing in the mouth and thus appears as a red viscous liquid (Figure 5b). The reconstituted liquid gel was subsequently introduced into an acidic medium (0.1 N HCl), where it transformed into a buoyant gel (Figure 5c). The resulting yellow, solidified gels exhibited sustained release behavior, thereby delaying the release of curcumin from the system (Figure 5d).

#### 2.2.2. Analysis of Texture Properties

Texture analysis is a widely applied technique for evaluating the mechanical properties of jelly products during deformation. The texture profiles of all formulations were assessed based on hardness, cohesiveness, springiness, gumminess, and chewiness, as presented in Table 1.

Significant differences were observed among the formulations. The hardness values indicated that increasing concentrations of KC and SA substantially enhanced gel firmness. This effect can be attributed to the ability of SA to form a chemically cross-linked network. In addition, the incorporation of κ-carrageenan acted as a structural reinforcer by physically intertwining with the alginate chains. This synergistic interaction resulted in a more robust double-network structure, which is critical for a chewable dosage form. All formulations exhibited relatively low cohesiveness; however, F5 showed the highest value among them. The springiness results suggested that most formulations maintained acceptable elasticity. Gumminess increased in parallel with hardness, reflecting the combined contribution of firmness and cohesiveness to the overall textural behavior. Similarly, chewiness was strongly influenced by both gumminess and hardness, with F1 exhibiting the lowest value and F12 the highest. Previous studies have reported that SA reinforces the KC gel matrix, resulting in enhanced gel strength and mechanical properties, including gumminess and chewiness [27]. Overall, both SA and KC played a crucial role in modulating the texture characteristics of the formulations.

### 2.3. Physical Properties of the Formed Gels

#### 2.3.1. Density and Buoyancy Lag Time of Formed Gels

The oral in situ gel-forming system consists of polymers combined with effervescent agents to enable buoyancy. Upon exposure to an acidic medium, an ion-triggered crosslinking process occurs as the liquid formulation interacts with the environment. This reaction promotes the release of Ca^2+^ ions from calcium carbonate (CaCO_3_) incorporated in the formulation. The liberated Ca^2+^ ions function as crosslinking agents, inducing the formation of an alginate-based gel network. Concurrently, carbon dioxide (CO_2_) is produced and becomes entrapped within the hydrogel matrix, thereby enhancing the floating capability of the system [6,28]. The density of all formulations was lower than that of gastric fluid (1.004 g/cm^3^). Consequently, they demonstrated rapid buoyancy following contact with the acidic medium, attributed to CO_2_ generation (Table 2).

#### 2.3.2. Gel Strength

Gastric compression forces are critical factors influencing the performance of gastroretentive dosage forms. In this study, SA at concentrations of 0.5%, 0.75%, and 1% was combined with varying levels of KC to investigate their effects on gel strength. The formulations were exposed to an acidic medium to induce gel formation, and the resulting gels were evaluated for mechanical strength.

As shown in Table 2, the gel hardness of the twelve formulations (F1–F12) varied significantly, with F1–F4 exhibiting low values, F5–F8 showing a marked increase, and F9–F12 demonstrating the highest hardness. F1 had the lowest gel strength, while F12 showed the highest. These findings indicate that modifications in polymer composition substantially influenced gel mechanical properties. The enhanced gel strength can be attributed to the crosslinking ability of alginate in the presence of Ca^2+^ ions. Calcium ions bind to guluronic acid (G) segments of alginate molecular chains, forming the characteristic “egg-box” structure that reinforces the three-dimensional gel network [20,29]. Upon exposure to acidic conditions, Ca^2+^ ions are released and promote intermolecular crosslinking, thereby improving the structural integrity and mechanical resistance of the gel matrix [21].

### 2.4. In Vitro Curcumin Release from Floating In Situ Gels

**Figure 6:**
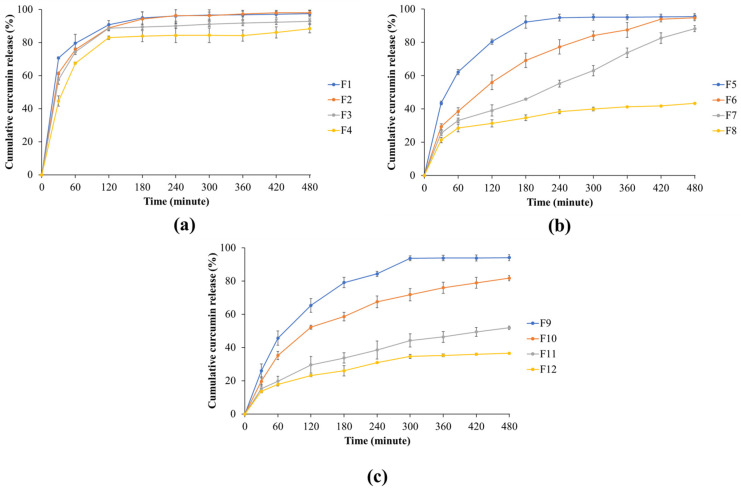
Curcumin release profiles of all formulations. (**a**) Effect of 0.5% SA mixed with 0.5–2% KC; (**b**) effect of 0.75% SA mixed with 0.5–2% KC; and (**c**) effect of 1% SA mixed with 0.5–2% KC.

The cumulative release behavior of curcumin formulations is illustrated in Figure 6. In the first group (0.5% SA combined with 0.5–2% KC), a rapid initial drug release was observed within the first 60 min (F1 > F2 > F3 > F4), with cumulative release exceeding 70%, followed by a plateau phase. As shown in Figure 6a, formulations F1–F3 achieved nearly complete release (>95%) within 240 min, whereas F4 exhibited a slightly lower release of approximately 90%.

The second group (0.75% SA with 0.5–2% KC) demonstrated more distinct differences in release behavior (Figure 6b). F5 showed the fastest and highest release, reaching nearly complete drug release within 240 min. In contrast, F6 and F7 exhibited moderate release rates, achieving approximately 90% and 80% release at 480 min, respectively. F8 presented the most sustained profile in this group, with cumulative release remaining below 50% throughout the study period. For the third group (1% SA with 0.5–2% KC; Figure 6c), variable release patterns were also observed. F9 displayed rapid release, reaching nearly 100% at approximately 300 min, while F10 achieved about 85% release at 480 min. F11 showed a slower release profile (~65% at 480 min), and F12 demonstrated the most prolonged release, not exceeding 40% during the test period.

The sustained release behavior of curcumin is attributable to the increased viscosity and strengthened gel matrix at higher polymer concentrations, which limit drug diffusion compared with lower-viscosity systems [21,25,28]. Overall, increasing the concentrations of both SA and KC effectively retarded curcumin release.

### 2.5. Cell Viability of RAW264.7 and 3T3-L1 Cells

**Figure 7:**
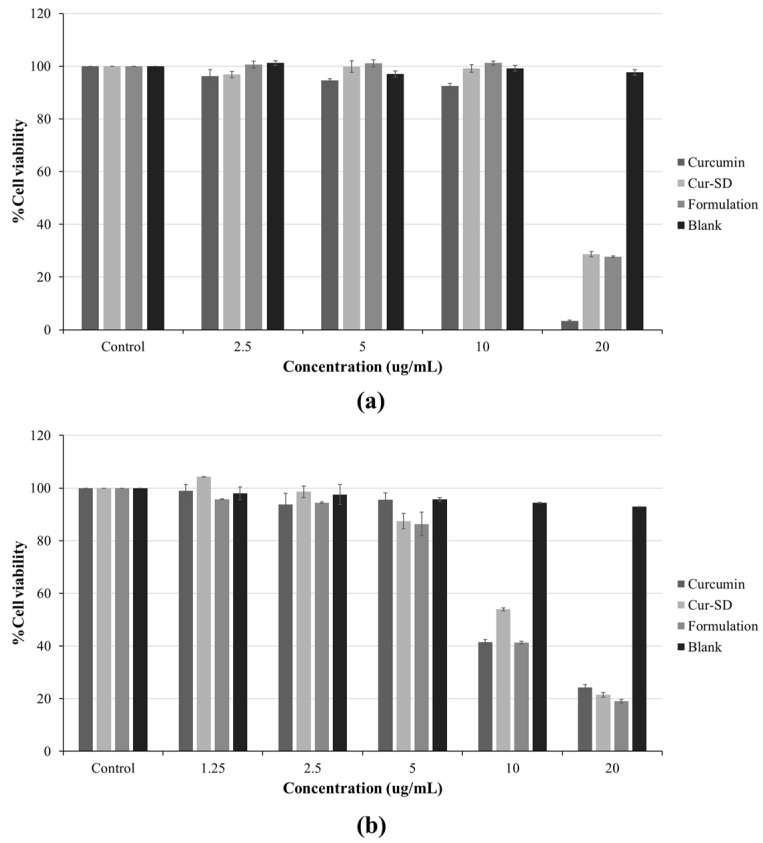
The cell viability of RAW264.7 macrophage cells (**a**) and 3T3-L1 adipocyte cells (**b**), followed by control, unformulated curcumin, CUR-SD (1:3), F7 formulation (equivalent to curcumin concentration), and F7 blank.

Cell viability of both RAW264.7 and 3T3-L1 cells was determined by MTT assay after 24 h of treatment. Cells were treated with four samples, including curcumin, CUR-SD (1:3), F7 formulation (equivalent to curcumin concentration), and F7 blank at concentrations of 20, 10, 5, 2.5 1.25 μg/mL. As shown in Figure 7, RAW264.7 macrophages maintained cell survival above 90% for all samples at concentrations up to 10 μg/mL (Figure 7a). By contrast, 3T3-L1 adipocytes exhibited cell viability above 90% at concentrations of 2.5 μg/mL (Figure 7b). Notably, the F7 blank, tested at concentrations equivalent to the dry weight of the formulation, presented high cell survival across all tested concentrations, indicating no cytotoxic effect. Based on these results, 5 μg/mL was selected as the maximum non-cytotoxic concentration suitable for subsequent anti-inflammatory assays and anti-obesity assays in both cell lines.

### 2.6. Anti-Inflammatory Activity

The anti-inflammatory activity of curcumin, CUR-SD (1:3), F7 formulation, and F7 blank was evaluated by assessing nitric oxide (NO) level in LPS-induced RAW264.7 macrophages. The inhibitory effects of all samples, as well as the reference drug indomethacin, were displayed as IC_50_ values in Table 3. Curcumin, CUR-SD (1:3), and F7 formulation demonstrated comparable inhibitory effects, with IC_50_ values ranging from 4.12 to 4.84 µg/mL. In comparison, the reference drug indomethacin showed markedly weaker inhibitory activity (IC_50_ = 46.35 + 0.45 µg/mL), while the F7 blank did not exhibit inhibitory activity against NO production.

### 2.7. Inhibitory Effects on Lipid Accumulation

**Figure 8:**
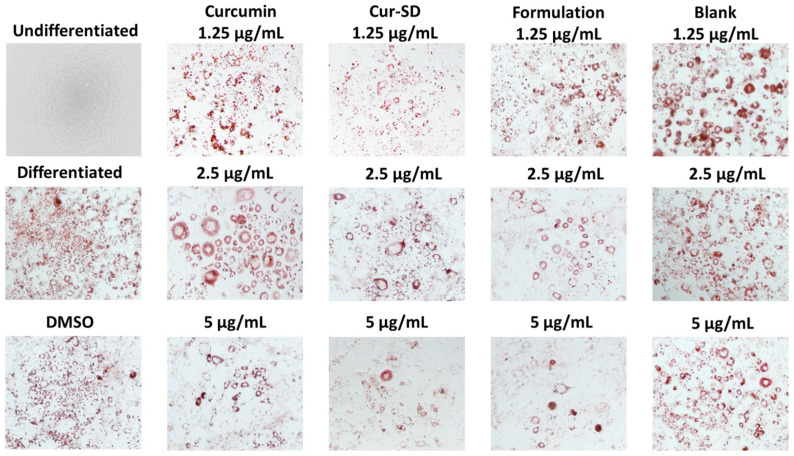
Oil Red O staining of lipid accumulation in 3T3-L1 adipocytes after 10 days of differentiation. 3T3-L1 cells were induced for differentiation and were exposed to 0.1% DMSO, curcumin, CUR-SD, the formulation, and the blank at concentrations of 1.25, 2.5, and 5 µg/mL. Undifferentiated 3T3-L1 pre-adipocytes were used as the undifferentiated control.

**Figure 9:**
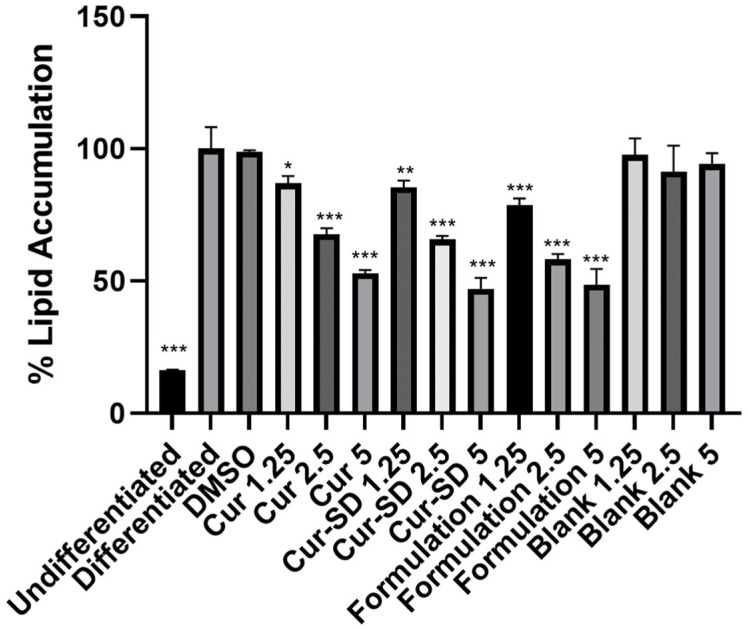
Quantification analysis of lipid accumulation in 3T3-L1 adipocytes. Lipid droplets in undifferentiated and differentiated 3T3-L1 adipocytes treated with 0.1% DMSO, Curcumin, Cur-SD, formulation, and corresponding blanks at concentrations of 1.25, 2.5, and 5 µg/mL were stained with Oil Red O. The dye was subsequently eluted with isopropanol and quantified spectrophotometrically at 520 nm. Results are expressed as a percentage relative to the differentiated control. Data are presented as mean + SD from three independent experiments. Statistical significance was determined in comparison with the differentiated control (* *p* < 0.05, ** *p* < 0.01, ***p < 0.0001).

Lipid accumulation in 3T3-L1 cells was visualized using Oil Red O staining after adipogenic induction for 10 days. As demonstrated in Figure 8 and Figure 9, undifferentiated cells exhibited minimal lipid accumulation (16.31 + 0.27% of lipid accumulation), whereas differentiated control cells showed extensive lipid droplet formation (100.06 + 8.09% of lipid accumulation), confirming successful adipocyte differentiation. The DMSO-treated vehicle control displayed a similar level of lipid accumulation (98.80 + 0.63% of lipid accumulation) to the differentiated control, indicating that DMSO did not interfere with the differentiation process.

Regarding the treatment effects, at the highest concentration (5 µg/mL), curcumin, CUR-SD, and the formulation significantly inhibited lipid accumulation by approximately 47–53%, whereas the formulation blank did not markedly alter lipid droplet formulation. Lower concentrations of curcumin-containing samples showed a similar trend with reduced magnitude of inhibition, indicating a clear dose-dependent effect.

Collectively, it confirmed that observed inhibitory effects of lipid accumulation were attributable to curcumin rather than the carrier system. Although CUR-SD and the formulation did not show stronger inhibition than free curcumin at the same concentrations, their comparable effects indicate that the formulation effectively maintained the biological activity of curcumin during adipogenic differentiation [30,31]. This finding is important considering the well-known limitations of curcumin, particularly its poor solubility and stability in aqueous environments [25]. While a clear increase in potency was not observed under the present in vitro conditions, the formulation may still provide practical benefits by ensuring consistent anti-adipogenic activity and facilitating curcumin delivery. Overall, these results support the use of formulation strategies to improve curcumin handling without compromising its inhibitory effect on lipid accumulation.

## 3. Conclusions

This study developed a gastroretentive chewable gel incorporating curcumin solid dispersion to overcome poor solubility and enhance therapeutic performance. Amorphous dispersion in Eudragit^®^ EPO significantly improved acidic dissolution, while the sodium alginate/κ-carrageenan system enabled rapid ion-triggered in situ gelation with suitable buoyancy, mechanical strength, and sustained release. Polymer concentration governed gel integrity and diffusion-controlled drug release. The optimized formulation (F7; 0.75% SA, 1.5% KC) demonstrated biocompatibility and retained anti-inflammatory and anti-obesity activities. Overall, this gastroretentive platform represents a promising strategy to enhance curcumin efficacy in obesity management.

## 4. Materials and Methods

### 4.1. Materials

Curcumin (96.1% *w*/*w*) was purchased from Thai–China Flavors and Fragrances Industry Co., Ltd. (Phra Nakhon Si Ayutthaya, Thailand). Sodium alginate (SA) (viscosity value ~2000 cPs of 2% *w*/*w* solution at 25 °C) was obtained from High Science Co., Ltd. (Songkhla, Thailand), and Kappa-carrageenan was purchased from Krungthepchemi Co., Ltd. (Bangkok, Thailand). Eudragit^®^ EPO was a gift from Jebsen & Jessen Ingredients (Bangkok, Thailand). Calcium carbonate was obtained from LOBA Chemie Pvt. Ltd. (Mumbai, India). Sodium bicarbonate was purchased from RCI Labscan (Bangkok, Thailand).

3T3-L1 (CL-173™, fibroblast cell line) and RAW264.7 (TIB-71™, murine macrophage cell line) were obtained from American Type Culture Collection (ATCC; Manassas, VA, USA). Lipopolysaccharide (LPS), dexamethasone (DEX), 3-isobutyl-1-methylxanthine (IBMX), Oil Red O (0.5% in isopropanol), insulin, indomethacin, and Griess reagent were purchased from Sigma-Aldrich (St. Louis, MO, USA). Dulbecco’s Modified Eagle’s Medium (DMEM), Roswell Park Memorial Institute 1640 medium (RPMI-1640), 3-(4,5-dimethyl-2-thiazolyl)-2,5-diphenyl-2H-tetrazolium bromide (MTT), phosphate-buffered saline (PBS), trypsin–EDTA (0.25%), trypan blue, and penicillin–streptomycin (PS) were obtained from Gibco (Invitrogen, CA, USA). Fetal bovine serum (FBS) was purchased from Cytiva (Bangkok, Thailand). Dimethyl sulfoxide was obtained from Amresco (Solon, OH, USA).

### 4.2. Preparation of Curcumin-Loaded Solid Dispersions

Curcumin solid dispersions (CUR-SD) were obtained via the solvent evaporation process with Eudragit^®^ EPO as the carrier at drug-to-polymer weight ratios of 1:1, 1:3, 1:5, and 1:7 [25,32]. Briefly, 1 g of curcumin was weighed and dissolved in 500 mL absolute ethanol. The appropriate amount of polymer was then added to the solution with continuous stirring until completely dissolved, and added to a round-bottom flask. Then, the flask containing the solution was processed in a rotary evaporator (Heidolph Instruments GmbH, Schwabach, Germany) to remove the solvent under reduced pressure at 40 °C for 6–10 h. The solid mass was milled using a glass mortar and pestle; the resulting powder was sieved to a particle size of 50–250 µm. Both CUR-SD and CUR-PM preparations were then kept in light-protected, airtight containers at ambient temperature.

### 4.3. Evaluation of Curcumin Solid Dispersions

#### 4.3.1. Analysis of Curcumin Contents

Curcumin contents were analyzed using a UV spectrophotometric technique with a spectrophotometer (UV-1900i, SHIMADZU Corporation, Kyoto, Japan) and measured at a wavelength of 425 nm compared to a curcumin standard curve [25]. The calibration curve was established using the standards of curcumin at a concentration in the range of 1 to 5 µg/mL (y = 0.137x + 0.0014, R^2^ = 0.9999). The measurements were performed in triplicate.

#### 4.3.2. Solubility Study of Curcumin Solid Dispersions

The aqueous solubility of free curcumin, CUR-PMs, and CUR-SDs at different ratios was evaluated using the shake-flask technique as previously reported [32]. In brief, an excess amount of each formulation was introduced into a 15 mL tube containing 1 mL of 0.1 N HCl (pH 1.2) and vortex-mixed (Vortex-Genie 2, 50 Hz model, Scientific Industries Inc., Bohemia, NY, USA) for 10 min. The suspensions were then incubated in a shaking water bath at 37 °C for 48 h to reach equilibrium. Following incubation, samples were centrifuged at 4000 rpm for 30 min at ambient temperature (Kubota 5922B/N, Kubota, Tokyo, Japan). The supernatant fractions were carefully retrieved, appropriately diluted with 0.1 N HCl, and passed through a 0.45 µm membrane filter prior to UV–visible spectrophotometric analysis as described in Section 4.3.1. The measurements were performed in triplicate.

#### 4.3.3. Dissolution Study of Curcumin Solid Dispersions

The dissolution profiles of free curcumin, CUR-PMs, and CUR-SDs were evaluated in 0.1 N HCl (pH 1.2) as described previously [25,32]. Accurately weighed samples were introduced into 900 mL of dissolution medium and tested using a USP type II apparatus (PTWS 120D, Pharma Test, Hainburg, Germany) operated at 50 rpm and kept at 37 ± 0.5 °C. At specific intervals (5, 10, 15, 30, 45, 60, 90, and 120 min), 5 mL aliquots were sampled and immediately replaced with an equivalent volume of fresh medium to ensure the maintenance of sink conditions. Each collected sample was passed through a 0.45 μm membrane filter before being quantified using a UV–visible spectrophotometric method.

#### 4.3.4. Physicochemical Characterization of Curcumin Solid Dispersion

Powder diffraction analysis

The crystallinity of samples was analyzed using powder X-ray diffraction (PXRD) of free curcumin, Eudragit^®^ EPO, physical mixtures, and solid dispersions. Each sample was ground using a mortar and pestle prior to analysis. PXRD spectra were assessed using an X-ray diffractometer (X’pert MPD, Philips, The Netherlands) at room temperature. Each sample was assessed under the following conditions: 40 kV, a current of 30 mA, a scan speed of 1 s/step over 2 theta, a scan range of 5–90°, and a step size of 0.05°.

Fourier Transform Infrared Spectroscopy

Fourier transform infrared (FT-IR) spectroscopy (Vertex 70, Bruker, Ettlingen, Germany) was employed to investigate the functional groups and potential intermolecular interactions between the drug and polymer in both the physical mixture and the solid dispersion. Each sample was blended with potassium bromide (KBr) using a mortar and pestle and subsequently compressed into pellets with a compaction press. Spectra were recorded over the wavenumber range of 400–4000 cm^−1^ at a resolution of 4 cm^−1^.

### 4.4. Formulation of Gummy Jellies Loaded with Curcumin-Solid Dispersion

Gummy jelly formulations were developed [22] using varying concentrations of sodium alginate (SA) and κ-carrageenan (KC), while the amounts of sodium benzoate, calcium carbonate, sucralose, and lemon flavor were kept constant, as presented in Table 4.

Briefly, SA and KC were dispersed in deionized water preheated to 80 °C and stirred under magnetic agitation until a clear solution formed. After complete dissolution, calcium carbonate was incorporated, and the total weight was adjusted to 100 g. The mixture was then cooled to 40 °C before adding the Cur-SD formulation. The resulting suspension was poured into silicone jelly molds (3 × 3 × 1.5 mm), with each unit weighing 10 g and containing an equivalent of 40 mg of curcumin per piece. The molded jellies were refrigerated at 4 ± 2 °C for 1 h to allow for gelation, then transferred to a sealed plastic container and stored at room temperature (25 ± 2 °C) for subsequent evaluation.

### 4.5. Evaluation of Gummy Jellies Loaded with Curcumin-Solid Dispersion

#### 4.5.1. Physical Appearance and pH

The physical appearance of the gummy jellies was assessed by visual inspection. The pH of the reconstituted jelly formulation was determined using a digital pH meter (Mettler-Toledo GmbH, Zurich, Switzerland) at 25 ± 1 °C. For pH measurement, 1 g of gummy jelly was precisely weighed and dispersed in 100 mL of deionized water preheated to 40 °C, followed by stirring until a homogeneous solution was obtained. All measurements were carried out in triplicate.

#### 4.5.2. Texture Profile Analysis of Gummy Jellies

The textural characteristics of the gummy jellies were characterized using a texture analyzer (TA.XT Plus, Stable Micro Systems, London, UK) fitted with a 25 mm diameter cylindrical probe [33]. Texture profile analysis mode was applied under the following conditions: pre-test speed of 1 mm s^−1^, test speed of 5 mm s^−1^, and post-test speed of 5 mm s^−1^. The compression distance corresponded to 50% strain, using a 5 kg load cell and a trigger force of 5 g. Key parameters, including hardness, cohesiveness, adhesiveness, springiness, gumminess, and chewiness, were calculated. All measurements were conducted in triplicate.

#### 4.5.3. Determination of Curcumin Contents in Gummy Jellies

A gummy jelly sample was cut into small pieces and extracted with 80 mL of methanol in a 100 mL volumetric flask [25]. The mixture underwent sonication in an ultrasonic water bath to ensure complete dispersion (Crest Ultrasonic Corp., Ewing Township, NJ, USA) for 30 min. After sonication, the solution was diluted to 100 mL with methanol and subsequently passed through a 0.45 µm membrane filter. Curcumin content was determined using UV–visible spectrophotometry at a wavelength of 425 nm. All measurements were conducted in triplicate.

### 4.6. Evaluation of Floating In Situ Gels

#### 4.6.1. Gel Density Measurement

The density of the gel was assessed using the procedure described by Fungfoung et al. 2023 [6]. Briefly, a glass cylinder containing 75 mL of 0.1 N HCl solution was weighed and recorded as W_1_. In a separate experiment, 10 g of the reconstituted gelling solution was placed into a 100 mL graduated cylinder and allowed to stand for 30 min to permit complete gel formation. After gelation, the combined weight of the graduated cylinder and gel was measured (W_2_). The volume of the formed gel (V) was directly obtained from the graduated scale. The gel density (ρ) was subsequently calculated using the following equation:

Density = W_2_ − W_1_/V (1)

#### 4.6.2. Gel Strength Measurement

Gel strength was assessed using a texture analyzer fitted with a cylindrical probe (25 mm diameter), following the procedure reported by Fungfoung et al. (2023) [6]. Gels were prepared directly in a 250 mL beaker containing 150 mL of 0.1 N HCl solution (pH 1.2) and maintained at 37 °C for 30 min to allow for complete gelation. The beaker with the formed gel was then placed on the heavy-duty platform of the instrument. The probe was lowered into the gel under the following conditions: pre-test speed of 1 mm s^−1^, test speed of 1 mm s^−1^, and post-test speed of 5 mm s^−1^. The compression force (g) required to penetrate the gel was recorded as an indicator of gel strength. All experiments were conducted in triplicate.

#### 4.6.3. Gel Floating Characteristics

The buoyancy of the formed gels was assessed by determining the buoyancy lag time and total floating duration using a USP type II dissolution apparatus containing 900 mL of 0.1 N HCl solution (pH 1.2) maintained at 37 °C [6,28]. The liquid formulation was introduced into the acidic medium, and the time required for the gel to rise to the surface (floating lag time) was recorded. The total floating time was defined as the duration during which the gel remained continuously buoyant on the surface of the medium. All experiments were conducted in triplicate.

### 4.7. Curcumin Release from Floating In Situ Gels

In vitro drug release was determined using a USP type II (paddle) dissolution apparatus following the method described by Siripruekpong et al. (2023) [25]. Dissolution was conducted in 900 mL of 0.1 N HCl (pH 1.2) maintained at 37 ± 0.5 °C, with a rotation speed of 50 rpm. A 20 g portion of the liquid gummy jelly formulation (which mimics the action of chewing in the mouth), pre-equilibrated to 37 °C, was introduced into the dissolution vessel to allow for in situ gel formation. Aliquots (5 mL) were sampled at designated intervals from 30 to 480 min. To ensure sink conditions and a constant dissolution volume, an equal volume of fresh, pre-warmed medium was added after each sampling. Curcumin concentration was determined by UV–visible spectrophotometry at 425 nm.

### 4.8. Evaluation of Biological Activities

#### 4.8.1. Cell Culture

RAW264.7 cells were cultured in RPMI-1640 medium containing 10% fetal bovine serum and 1% PS solution. 3T3-L1 cells were grown in DMEM enriched with 10% fetal bovine serum and 100 U/mL penicillin, and 100 µg/mL streptomycin. Both cell lines were maintained at 37 °C in a 5% CO_2_ incubator. The cultured medium was replaced every two days and the cells were subcultured once they reached 70% confluence.

#### 4.8.2. Cell Viability Assay

The cytotoxic effects of samples were evaluated in RAW264.7 and 3T3-L1 cells using the MTT assay [28,34]. RAW264.7 or 3T3-L1 cells were cultured in 96-well plates at densities of 1 × 10^5^ and 1 × 10^4^ cells/well, respectively. The cells were allowed to attach overnight and then treated with four samples of various concentrations (6.25, 12.5, 25, 50, and 100 μg/mL), including curcumin, cur-SD, formulation (equivalent doses of the extracted curcumin), and blank, for 24 h. After incubation, the treatment medium was renewed with fresh medium containing 0.5 mg/mL of MTT solution, followed by further incubation for 3 h. Subsequently, the MTT-treated medium was removed, followed by the addition of DMSO to dissolve the formazan crystals. The absorbance was measured at 570 nm using the microplate reader (SPECTRO star Nano, Ortenberg, Germany). Cell viability (%) was determined using the following formula (Equation (2));

% Cell viability = [ODs/ODc] × 100(2)

where ODs and ODc are the absorbance at 570 nm of the sample and control, respectively.

#### 4.8.3. Determination of Anti-Inflammatory Activity

The anti-inflammatory activity of the samples was determined using an LPS-stimulated RAW264.7 macrophage model [28,35]. Briefly, RAW264.7 cells were cultured in the 96-well plate at a density of 1 × 10^5^ cells/well and maintained at 37 °C in a humidified atmosphere with 5% CO_2_ for 10–12 h. Then, the cells were treated with different concentrations of samples with or without LPS (at a final concentration of 100 ng/mL). After incubation for 24 h, the supernatant fractions were transferred into a new plate and mixed with the Griess reagent at a 1:1 ratio. The absorbance was then measured at 570 nm. The inhibitory percentage of nitric oxide production was calculated relative to the LPS-treated control.

#### 4.8.4. Determination of Anti-Obesity Activity

3T3-L1 adipocyte differentiation

3T3-L1 preadipocyte cells were differentiated into mature adipocytes according to the protocol described by Fungfoung et al. (2025) [9]. Cells were seeded in 48-well plates at a density of 2 × 10^4^ cells per well and cultured in complete growth medium for four days, with medium replacement every 48 h. Upon reaching full confluence, adipogenic differentiation was initiated using an induction medium composed of complete medium supplemented with 1 µM dexamethasone, 0.5 mM IBMX, and 10 µg/mL insulin for 48 h. The induction medium was subsequently renewed with maintenance medium containing complete medium and 10 µg/mL insulin. The maintenance medium was refreshed every 48 h for 6–8 days to allow for the full maturation of adipocytes.

Lipid Accumulation by Oil Red O staining

The inhibitory effect of the samples on lipid accumulation was determined by Oil Red O staining, following a modified protocol of Fungfoung et al. (2025) [9]. 3T3-L1 cells were differentiated as previously described and treated with various concentrations of the samples throughout the differentiation period. After induction, cells were maintained for 8 days in maintenance medium containing the respective treatments. Upon completion of differentiation, cells were rinsed with PBS, fixed with 10% formalin for 2 h, rinsed with 60% isopropanol, and dried at room temperature. Cells were treated with Oil Red O for 10 min and rinsed to remove excess dye. Lipid droplets were visualized using an inverted microscope (Nikon ECLIPSE Ts2, Tokyo, Japan). For quantification, the dye was eluted with isopropanol, and absorbance was measured at 520 nm. Lipid accumulation was expressed as a percentage relative to the differentiated untreated control group.

### 4.9. Statistical Analysis

All values are expressed as mean ± standard deviation (S.D.). Statistical comparisons were made using Student’s *t*-test and one-way ANOVA. Statistical probability (*p*) values < 0.05 were considered to denote significant differences.

## Figures and Tables

**Table 1:** Texture profile analysis of all formulations.

Formulations	Texture Analysis				
	Hardness (g)	Cohesiveness	Springiness (mm)	Gumminess (g)	Chewiness (g)
F1 (SA0.5/KC0.5)	61.36 ± 3.22	0.138 ± 0.00	0.792 ± 0.05	8.45 ± 0.41	6.73 ± 0.73
F2 (SA0.5/KC1)	203.07 ± 4.67 ^a^	0.123 ± 0.00	0.696 ± 0.12	25.05 ± 0.99 ^a^	19.12 ± 1.38 ^a^
F3 (SA0.5/KC1.5)	251.61 ± 6.77 ^a^	0.113 ± 0.00	0.483 ± 0.02	28.34 ± 0.54 ^a^	21.80 ± 1.40 ^a^
F4 (SA0.5/KC2)	552.04 ± 4.62 ^a^	0.102 ± 0.00	0.715 ± 0.12	56.49 ± 1.19 ^a^	46.73 ± 0.61 ^a^
F5 (SA0.75/KC0.5)	66.65 ± 1.81	0.154 ± 0.00	0.838 ± 0.00	10.28 ± 0.08	8.62 ± 0.06
F6 (SA0.75/KC1)	259.88 ± 2.92 ^b^	0.124 ± 0.01	0.855 ± 0.00	32.21 ± 3.26 ^b^	27.55 ± 2.85 ^b^
F7 (SA0.75/KC1.5)	370.88 ± 8.16 ^b^	0.100 ± 0.01	0.851 ± 0.00	36.95 ± 1.94 ^b^	31.46 ± 1.61 ^b^
F8 (SA0.75/KC2)	728.31 ± 13.50 ^b^	0.086 ± 0.00	0.845 ± 0.00	62.37 ± 1.21 ^b^	52.72 ± 0.95 ^b^
F9 (SA1/KC0.5)	102.92 ± 2.80	0.133 ± 0.00	0.855 ± 0.04	34.24 ± 1.14	11.72 ± 0.85
F10 (SA1/KC1)	282.71 ± 2.93 ^c^	0.141 ± 0.01	0.837 ± 0.02	39.86 ± 1.74 ^c^	33.37 ± 1.96 ^c^
F11 (SA1/KC1.5)	446.33 ± 24.99 ^c^	0.111 ± 0.00	0.892 ± 0.00	49.51 ± 2.35 ^c^	44.18 ± 2.17 ^c^
F12 (SA1/KC2)	871.94 ± 9.20 ^c^	0.082 ± 0.00	0.830 ± 0.04	71.77 ± 1.81 ^c^	59.54 ± 1.57 ^c^

^a^ Statistically significant differences were accepted at *p* values < 0.05 when F1 was compared to F2–4 formulations. ^b^ Statistically significant differences were accepted at *p* values < 0.05 when F5 was compared to F5–8 formulations. ^c^ Statistically significant differences were accepted at *p* values < 0.05 when F9 was compared to F9–12 formulations.

**Table 2:** Physical properties of all formulations in liquid form.

Formulations	Floating Time (s)	Density (g/cm^3^)	Gel Strength (g)
F1 (SA0.5/KC0.5)	21.0 ± 3.00	0.873 ± 0.05	42.0 ± 1.03
F2 (SA0.5/KC1)	30.0 ± 1.00 ^a^	0.819 ± 0.03	69.0 ± 1.58 ^a^
F3 (SA0.5/KC1.5)	112.0 ± 4.36 ^a^	0.728 ± 0.04	98.3 ± 3.24 ^a^
F4 (SA0.5/KC2)	125.0 ± 6.00 ^a^	0.697 ± 0.03	170.2 ± 2.68 ^a^
F5 (SA0.75/KC0.5)	90.0 ± 2.65	0.788 ± 0.01	136.3 ± 2.50
F6 (SA0.75/KC1)	131.7 ± 3.21 ^b^	0.715 ± 0.03	223.8 ± 1.48 ^b^
F7 (SA0.75/KC1.5)	144.3 ± 0.58 ^b^	0.704 ± 0.03	332.0 ± 90.6 ^b^
F8 (SA0.75/KC2)	164.0 ± 2.65 ^b^	0.682 ± 0.01	544.2 ± 11.61 ^b^
F9 (SA1/KC0.5)	169.3 ± 1.53	0.716 ± 0.01	212.6 ± 6.10
F10 (SA1/KC1)	182.7 ± 3.51 ^c^	0.687 ± 0.03	301.6 ± 5.96 ^c^
F11 (SA1/KC1.5)	194.0 ± 2.65 ^c^	0.675 ± 0.03	411.6 ± 5.03 ^c^
F12 (SA1/KC2)	214.6 ± 2.0.8 ^c^	0.659 ± 0.02	645.4 ± 18.00 ^c^

^a^ Statistically significant differences were accepted at *p* values < 0.05 when F1 was compared to F2–4 formulations. ^b^ Statistically significant differences were accepted at *p* values < 0.05 when F5 was compared to F5–8 formulations. ^c^ Statistically significant differences were accepted at *p* values < 0.05 when F9 was compared to F9–12 formulations.

**Table 3:** Anti-inflammatory activity of curcumin and its related formulations.

Samples	Anti-Inflammatory Activity (NO Assay) IC_50_ (µg/mL)
Curcumin	4.12 ± 0.66 ^a^
CUR-SD	4.54 ± 0.39 ^a^
F7 formulation	4.84 ± 0.59 ^a^
F7 blank	N/A
Indomethacin	46.35 ± 0.45

^a^ Statistically significant differences were accepted at *p* < 0.05 of curcumin and its formulation compared with indomethacin. N/A = not active.

**Table 4:** Compositions of functional gummy jelly formulations.

Formulations	Variation in Ingredients (g)					
	Sodium Alginate	KC	Sodium Benzoate	CaCO_3_	Sucralose	Lemon Flavor Liquid
F1	0.50	0.50	0.10	1.50	0.20	1.00
F2		1.0				
F3		1.5				
F4		2.0				
F5	0.75	0.50	0.10	1.50	0.20	1.00
F6		1.0				
F7		1.5				
F8		2.0				
F9	1.00	0.50	0.10	1.50	0.20	1.00
F10		1.0				
F11		1.5				
F12		2.0				

Cur_SD was added equivalent to 400 mg of curcumin, and all formulations were adjusted to 100 g.

## Data Availability

Data will be made available on request.

## Abbreviations

ASDs	Amorphous solid dispersions
BMI	Body mass index
C/EBPs	CCAAT/enhancer-binding proteins
Ca^2+^	Calcium ion
CaCO_3_	Calcium carbonate
CO_2_	Carbon dioxide
CUR-PMs	Curcumin physical mixtures
CUR-SDs	Curcumin solid dispersions
DMSO	Dimethyl Sulfoxide
F	Formulation
FT-IR	Fourier-Transform Infrared Spectroscopy
G	Guluronic acid
g	Gram
g/cm^3^	Grams per cubic centimeter
HCl	Hydrochloric acid
IC	Inhibitory concentration
KC	κ-carrageenan
LPS	Lipopolysaccharide
MCP-1	Monocyte chemoattractant protein-1
mm	Millimeter
MTT	3-(4,5-dimethylthiazol-2-yl)-2,5-diphenyltetrazolium bromide
N/A	Not active
NAFLD	Non-alcoholic fatty liver disease
NF-κB	Nuclear Factor kappa-B
NO	Nitric oxide
PAI-1	Plasminogen activator inhibitor-1
PPARγ	Peroxisome proliferator-activated receptor gamma
SA	Sodium alginate
s	Second
TNF-α	Tumor Necrosis Factor-alpha
XRPD	X-ray powder diffraction
